# LogPpred: An AI-Based Predictive Model for Accurate Estimation of Molecular LogP

**DOI:** 10.3390/molecules31152566

**Published:** 2026-07-23

**Authors:** Lisa Piazza, Lara Sortino, Alessio Costa, Clarissa Poles, Federico Fornaseri, Stefano Sainas, Marta Giorgis, Giulio Poli, Marco Macchia, Marco L. Lolli, Tiziano Tuccinardi, Miriana Di Stefano

**Affiliations:** 1 Department of Pharmacy, University of Pisa, 56126 Pisa, Italy; lisa.piazza@phd.unipi.it (L.P.); l.sortino1@studenti.unipi.it (L.S.); a.costa14@student.unisi.it (A.C.); giulio.poli@unipi.it (G.P.); marco.macchia@unipi.it (M.M.); miriana.distefano@farm.unipi.it (M.D.S.); 2 Department of Life Sciences, University of Siena, 53100 Siena, Italy; 3 Genomics and Experimental Medicine Program, Scuola Superiore Meridionale (SSM, School of Advanced Studies), Via Mezzocannone 4, 80078 Napoli, Italy; c.poles@ssmeridionale.it; 4 Department of Drug Science and Technology, University of Turin, via Pietro Giuria 9, 10125 Turin, Italy; federico.fornaseri@unito.it (F.F.); stefano.sainas@unito.it (S.S.); marta.giorgis@unito.it (M.G.); marco.lolli@unito.it (M.L.L.); 5 Consorzio Interuniversitario Nazionale per la Scienza e Tecnologia dei Materiali (INSTM), 50121 Firenze, Italy

**Keywords:** lipophilicity, LogP prediction, machine learning, applicability domain, medicinal chemistry, ADMET, drug discovery

## Abstract

Lipophilicity, commonly described by the n-octanol/water partition coefficient (LogP), is a key physicochemical property influencing the pharmacokinetic behavior of small molecules. Reliable LogP estimation during the early stages of drug discovery is essential to support molecular design and prioritize compounds with favorable ADMET properties. In this work, we report the development of LogPpred, an AI-based predictor of molecular lipophilicity. Starting from a curated dataset of 13,536 molecules with experimentally determined LogP values, multiple machine learning algorithms and molecular representations were systematically evaluated. The best-performing model, based on Gaussian Process Regression and RDKit molecular descriptors, achieved a mean absolute error (MAE) of 0.34 on the independent internal test set. External validation on a fully independent OECD-derived dataset yielded an MAE of 0.84, demonstrating good generalization capability across diverse chemical space. Furthermore, experimental LogP determination of an additional set of independently selected compounds confirmed the predictive reliability of the model, yielding an MAE of 0.66. Comparative analyses showed that LogPpred outperformed several widely used LogP prediction tools. Applicability domain analysis further supported the reliability of the model, with MAE values improving to 0.28 and 0.62 for in-domain compounds in the internal and external validation sets, respectively. Overall, LogPpred represents a robust, accurate, and transparent tool for the early assessment of molecular lipophilicity in medicinal chemistry and drug discovery.

## 1. Introduction

The discovery and development of new bioactive molecules is a complex, resource-intensive process characterized by high attrition rates and substantial financial investment. The early identification of compounds with suitable pharmacokinetic and toxicological profiles is therefore essential to increase the likelihood of successful clinical translation and to reduce the overall time and cost associated with bringing new drugs to market [1,2]. Among the physicochemical parameters that most critically influence a molecule’s ADMET behavior, lipophilicity plays a central role, as it affects membrane permeability, solubility, metabolic stability, tissue distribution, and potential toxicity [3]. Lipophilicity is commonly quantified through the n-octanol/water partition coefficient (LogP), a widely adopted measure of a compound’s propensity to partition between hydrophobic and aqueous environments [4]. Given its broad relevance to pharmacokinetic processes, LogP has become a central descriptor in drug-likeness assessments, as reflected in Lipinski’s rule of five [5], in which an appropriate lipophilicity balance is recognized as critical for oral absorption and overall favorable pharmacokinetic behavior. In specific therapeutic contexts, such as compounds targeting the central nervous system, the optimal lipophilicity window differs, requiring careful modulation to enable efficient passage across the blood–brain barrier [6]. Consequently, obtaining a reliable estimate of LogP during the earliest stages of drug design is crucial to guide molecular optimization and reduce costly downstream failures. Experimental determination of LogP is traditionally performed using techniques such as shake-flask or HPLC, which provide accurate measurements but are limited by long processing times, consumption of materials, specialized instrumentation, and potential interlaboratory variability [7]. These constraints are incompatible with the high-throughput demands of modern drug discovery pipelines, emphasizing the need for fast and reliable computational approaches capable of supporting or partially replacing experimental determinations.

Several in silico methods have been developed over the years to estimate LogP, including fragment-based models (e.g., KowWIN, KLOGP, CLOGP), atom-based approaches (e.g., ALOGP, ALOGP98, XLOGP2/3), and methods relying on topological descriptors or computed physicochemical properties (e.g., MLOGP, TLOGP) [8,9,10,11,12,13,14]. While these approaches represent important milestones in computational chemistry, they inherently rely on predefined fragments or additive schemes and may struggle to capture the nonlinear and multifactorial contributions governing lipophilicity, especially across large and structurally diverse chemical spaces. These limitations can hinder predictive accuracy and restrict their applicability to broad and heterogeneous datasets.

The rise of machine learning (ML) has significantly transformed the landscape of cheminformatics, introducing powerful computational strategies capable of learning complex structure–property relationships from large and heterogeneous datasets [15]. ML algorithms can integrate multiple molecular representations, such as numerical descriptors and structural fingerprints (FPs), and detect nonlinear patterns that traditional QSAR approaches often fail to capture [16]. Beyond physicochemical property estimation, ML has become increasingly central in modern drug discovery pipelines, supporting tasks such as virtual screening, biological activity prediction, and the assessment of toxicity and ADMET profiles [17,18,19,20,21]. These advances have enabled the development of predictive models with enhanced generalizability, robustness, and accuracy across diverse chemical classes, thereby expanding the scope and impact of computational methods in early-stage drug design.

Despite these advances, the development of accurate, transparent, and computationally efficient models for LogP prediction remains an open challenge. Such models must combine methodological rigor with ease of use and wide applicability, allowing medicinal chemists to rapidly screen large molecular libraries and prioritize compounds with favorable lipophilicity profiles. The availability of reliable LogP prediction tools can substantially reduce the number of molecules requiring synthesis and experimental evaluation, thereby accelerating early-stage decision-making in drug design. In this context, we herein report the development of LogPpred, an ML model designed to accurately predict the lipophilicity of small molecules, expressed as the octanol/water partition coefficient (LogP). By leveraging a large number of experimentally determined LogP values and employing a diverse set of finely tuned ML algorithms and molecular representations, we succeeded in generating a highly reliable predictive framework. Furthermore, an applicability domain (AD) analysis was performed to define clear reliability boundaries for the predictions, thereby enhancing confidence in the model’s use for structurally diverse compounds.

Throughout the development of LogPpred, we aim to provide medicinal chemists and researchers in drug discovery with a valuable computational tool for the early assessment of molecular lipophilicity.

## 2. Results and Discussion

### 2.1. Model Generation, Optimization, and Evaluation

**Figure 1:**
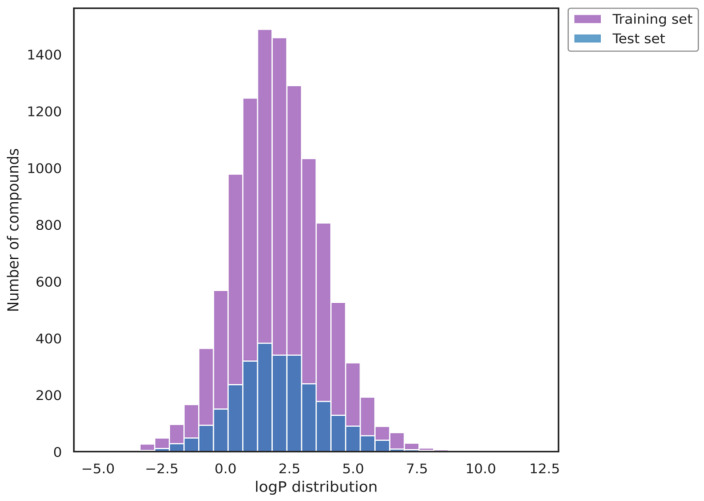
Distribution of experimental LogP values for the molecules in the training and test sets.

Lipophilicity, commonly quantified through the octanol/water partition coefficient (LogP), is a fundamental physicochemical property that governs membrane permeability, absorption, distribution, and, more broadly, the overall ADMET profile of small molecules. As LogP quantifies the balance between hydrophilic and hydrophobic tendencies, its accurate estimation is crucial for guiding early-stage molecular design and assessing drug-likeness. To establish a robust computational framework for accurate LogP predictions, we constructed a curated and chemically consistent dataset for the development and assessment of a broad panel of ML models. To this end, all curated LogP data were assembled into a unified dataset through a filtering and standardization workflow (see Section 3 for details), yielding a final collection of 13,536 unique molecules. This dataset was then split into a training set (80%) and an independent test set (20%), ensuring that no structural duplicates or charged species were introduced into model construction. In line with best practices for regression modeling, we verified that the training and test sets exhibited comparable chemical spaces and response distributions. Principal component analysis (PCA) of the dataset showed a substantial overlap between training and test sets, as illustrated in Figure S1, indicating that the former adequately captured the overall structural variability of the entire curated dataset, and that the latter did not contain isolated clusters or regions of chemical space absent in the training set. Furthermore, as shown in Figure 1, both subsets displayed nearly identical LogP distribution ranges and variances, implying that the partitioning procedure preserved the underlying statistical properties of the experimental measurements. Collectively, these analyses confirmed the suitability of the test set as a representative benchmark for evaluating the generalization ability of the proposed predictive models, ensuring that the reported performance metrics reflected true predictive capability rather than artifacts of dataset composition. Once the robustness and representativeness of the training/test partition had been established, a comprehensive set of predictive models was developed. For each compound, four distinct molecular representations were computed, capturing complementary layers of structural and physicochemical information. For traditional ML approaches, four molecular representations were computed for each compound, i.e., Morgan FPs, RDKit FPs, PubChem FPs, and RDKit Descriptors. These fixed-length representations were combined with five ML algorithms, namely Random Forest Regressor (RFR), Support Vector Regression (SVR), K-Nearest Neighbors Regressor (KNR), Gaussian Process Regression (GPR), and Multilayer Perceptron (MLP), resulting in a total of 20 distinct model configurations (see Section 3 for details).

In parallel, deep learning models were investigated using representation learning paradigms, in which molecular features were learned directly from raw molecular encodings rather than predefined descriptors. In this context, ChemBERTa was employed to derive molecular representations from canonical SMILES strings, which were processed using the pre-trained deepchem/ChemBERTa-77M-MTR model available through the Hugging Face model hub. This model is based on the RoBERTa transformer architecture and was trained on approximately 77 million chemically diverse compounds. For each molecule, the embedding corresponding to the [CLS] token from the final hidden layer was extracted and used as a fixed-length continuous representation. Owing to the scale and diversity of its pre-training corpus, ChemBERTa captures global and chemically meaningful molecular representations, encoding latent structural and physicochemical patterns that extend beyond predefined fragments or handcrafted descriptors. As a complementary approach, a convolutional graph neural network (cGNN) was used to learn molecular representations from graph-based encodings, where molecules are modeled as graphs with atoms and bonds as nodes and edges, respectively. Through message-passing operations, the cGNN aggregates atom-level information from local chemical environments, explicitly capturing molecular connectivity and topological relationships. Together, these two deep learning approaches provide alternative representation learning strategies that differ fundamentally from traditional feature-based models, enabling a direct comparison between handcrafted and learned molecular representations.

All models were optimized according to the procedures described in the Section 3, ensuring robust and reliable evaluation of predictive behavior across different data partitions. Model performance was assessed using the mean absolute error (MAE), with lower values indicating higher predictive accuracy and better agreement between predicted and experimental LogP values. The results, reported in Table S1 of the Supporting Information, provided clear insights into the influence of molecular descriptors and FP-based representations on the quality of LogP predictions. Overall, models developed using RDKit molecular descriptors consistently achieved lower prediction errors than their FP-based counterparts, indicating that continuous physicochemical features are more effective in capturing the molecular determinants of lipophilicity. Among the descriptor-based models, GPR delivered the best cross-validated performance, yielding the lowest MAE (0.34 ± 0.01), followed by SVR (0.38 ± 0.01), MLP (0.44 ± 0.01), and RFR (0.48 ± 0.01). These findings highlight the strong predictive value of descriptor-based representations across different ML algorithms. In contrast, FP-based models generally exhibited higher prediction errors. Among the evaluated FP representations, PubChem FPs provided the most consistent predictive performance, with MAE values ranging from 0.60 ± 0.02 to 0.70 ± 0.01 across different algorithms. Morgan FPs produced more variable results: although the SVR model achieved the lowest MAE among FP-based approaches (0.55 ± 0.01), the remaining Morgan-based models displayed considerably higher prediction errors, reaching up to 0.82 ± 0.02. Models developed using RDKit FPs also showed limited predictive accuracy, with MAE values ranging from 0.64 ± 0.01 to 0.75 ± 0.01. Overall, these results suggest that, although FP-based representations can provide reasonable predictive performance, they are generally less effective than molecular descriptors in capturing the subtle structural features governing LogP. In parallel, deep learning-based models were evaluated within the same CV described in the Section 3. The transformer-based ChemBERTa model achieved strong predictive performance, with an average MAE of 0.50 ± 0.03, ranking among the best-performing approaches and outperforming most FP-based models. In contrast, the cGNN exhibited more limited cross-validated performance, yielding an average MAE of 0.76 ± 0.01. Upon hyperparameter optimization and CV, all optimized models were trained on the full training set and applied to the internal test set derived from the random split of the curated dataset. For each model, predicted LogP values were compared with the corresponding experimental measurements, and predictive performance was evaluated in terms of both MAE and coefficient of determination (R^2^) (Table 1). The evaluation of all optimized models on the internal test set confirmed the trends observed during CV. Models trained on RDKit molecular descriptors consistently delivered the most accurate and stable predictions, with test set MAE values between 0.34 and 0.49 and R^2^ values between 0.85 and 0.92. In particular, the descriptor-based GPR model achieved the best overall performance, yielding the lowest prediction error (MAE = 0.34) and the highest correlation with experimental data (R^2^ = 0.92). PubChem FPs-based models showed intermediate predictive performance, with MAE values between 0.43 and 0.67 and R^2^ values ranging from 0.72 to 0.88, whereas Morgan and RDKit FPs-based models generally resulted in lower accuracy and weaker correlations. These findings indicate a reduced ability of binary FPs encodings to capture the structural determinants governing LogP. From an algorithmic perspective, descriptor-based GPR, SVR, and MLP models consistently achieved the most favorable balance between prediction accuracy and correlation with experimental values. On the internal test set, the ChemBERTa-based model achieved competitive predictive performance (MAE = 0.46, R^2^ = 0.88), confirming that large-scale molecular language models can capture global structural and physicochemical patterns relevant to lipophilicity directly from sequence-based representations. Nevertheless, despite its strong performance, ChemBERTa did not outperform the best descriptor-based configurations. The cGNN-based model achieved an MAE of 0.65 and an R^2^ of 0.73, remaining less accurate than the top-performing models. This outcome is likely associated with the relatively limited size of the training dataset, as graph-based deep learning models typically require large and diverse datasets to fully exploit their representational capacity when trained from scratch. Based on these results, the descriptor-based GPR model was retained as the final predictor and is hereafter referred to as LogPpred. To further assess the generalization ability of the selected model, we employed an external validation set which contains experimentally determined n-octanol/water partition coefficients. This dataset, retrieved from OECD eChemPortal and subjected to the same hierarchical curation workflow applied to the primary data (see Section 3 for details), resulted in 652 unique molecules with associated experimental LogP values obtained according to the OECD Test No. 107 guideline.

Importantly, none of these compounds were included in the training nor in the internal test set, ensuring that this dataset served as a fully independent benchmark for external evaluation. LogPpred was applied to all compounds in this dataset, and its predictions were compared with the corresponding experimental values using MAE as a measure of average prediction error. In this evaluation, LogPpred achieved an MAE of 0.84, indicating that the model was able to capture relevant lipophilicity trends even across a structurally diverse set of molecules spanning a broad LogP range (−4.45 to 11.66). As expected for an external validation scenario involving heterogeneous chemical space, a decrease in performance was observed relative to the internal test set. Nonetheless, the accuracy obtained remains consistent with robust predictive behavior and demonstrates that LogPpred retains meaningful generalization capability when applied to previously unseen data originating from independent regulatory sources.

### 2.2. Applicability Domain

To better define the reliability of LogPpred across diverse regions of the chemical space, the AD was assessed using ADvisor, a freely available tool for AD exploration and definition [22]. ADvisor provides a standardized and reproducible framework for AD estimation by benchmarking multiple methodological families and identifying the most suitable approach for a given model. Among the AD strategies evaluated, including distance-based, leverage-based, fragment-based, geometric, density-based, and ML approaches, the Composite AD (ComAD) method emerged as the most appropriate to define the AD for LogPpred. The ComAD strategy is a modular framework developed to delineate the reliability boundaries of molecular predictive models. Unlike conventional methodologies that often rely on restricted, single-metric criteria, ComAD integrates four orthogonal indices into a unified score to reflect a compound’s position within the modeled space. This multifaceted approach combines global chemical similarity and substructural analysis with dynamic measures of model behavior, such as the concordance between query predictions and neighbor experimental values, as well as the local predictive accuracy observed on similar test instances. By aggregating these diverse parameters, the strategy provides a transparent and robust mechanism for ensuring the scientific rigor and regulatory validity of in silico assessments. Within this framework, molecules falling inside the defined boundary are considered in-domain, whereas those that lie outside are classified as out-of-domain and are associated with reduced prediction reliability. Upon the identification of ComAD (threshold = 0.6, a = 1, b = 0.5, c = 0.5, d = 1; we refer the reader to the original reference [22] for a detailed description of these parameters) as the most suitable AD strategy, this was first applied to the internal test set derived from the 80/20 split of the curated dataset. Of the 2708 molecules in the internal test set, 2224 were classified as in-domain and 484 as out-of-domain. A clear difference in predictive accuracy was observed between these two subsets: for in-domain molecules, the model achieved an MAE of 0.28, whereas the MAE increased to 0.60 for out-of-domain molecules. These results demonstrate that LogPpred provides highly reliable predictions when operating within the chemical environments represented during training, while accuracy slightly decreases for molecules distant from the training space. The identified AD approach was subsequently applied to label the external validation set derived from OECD eChemPortal. Among the 652 curated molecules, 342 were classified as in-domain and 310 as out-of-domain. For in-domain molecules, LogPpred reached an MAE of 0.62, while this score increased up to 1.08 for out-of-domain compounds, confirming at the same time an expected modest decline in predictive accuracy for external predictions and highlighting the importance of defining the AD, which allows the identification of molecules associated with higher predictive error. Overall, these findings demonstrate that LogPpred provides robust and reliable predictions for molecules residing within its learned chemical space, while the AD annotation offers a transparent mechanism for identifying compounds for which prediction uncertainty is substantially higher. The combined use of LogPpred and its AD therefore enhances model interpretability and supports responsible deployment in early-stage drug discovery.

### 2.3. Comparison with Existing LogP Prediction Tools

To evaluate the effectiveness of our approach in a broader, practically relevant context, we compared the performance of LogPpred against a selection of widely used LogP prediction tools that represent the principal computational paradigms currently adopted in cheminformatics. These include fragment-based models, atom-based QSAR approaches, topological predictors, and modern deep-learning frameworks, many of which are routinely used in early-stage drug discovery and regulatory assessment. The first group of reference tools comprises the VEGA QSAR suite [23], which incorporates several well-established QSAR models for LogP prediction, including the Meylan/Kowwin, AlogP and MLogP models. The Meylan/Kowwin model implements the classical fragment-contribution method in which a molecule is decomposed into predefined fragments, each associated with a hydrophobicity coefficient, corrected by structural factors reflecting intramolecular interactions [9]. The ALogP model is based on the Ghose–Crippen–Viswanadhan atomistic approach, in which each atom type contributes additively to the estimated lipophilicity. The MLogP method relies on a multiple linear regression equation using thirteen topological descriptors. These QSAR models are widely employed in regulatory domains due to their simplicity, mechanistic interpretability, and long-standing use in chemical risk assessment. We also considered modern ML platforms such as Deep-PK [24] and ADMET-AI [25]. Deep-PK predicts LogP using direct message passing neural networks (DMPNNs) derived from ChemProp, integrating learned graph-based features with RDKit descriptors. By contrast, ADMET-AI is an integrated platform for multi-endpoint ADMET prediction based on deep-learning approaches, which estimates lipophilicity through an atom-based LogP calculation derived from Crippen’s method as implemented in RDKit. Another widely used platform by the scientific community is SwissADME [26], a web-based resource offering a broad range of physicochemical and pharmacokinetic estimations, including several LogP prediction models grounded in different theoretical approaches (e.g., atomistic, fragment-based, and topological methods). Additionally, SwissADME computes a consensus value considering all the predictions returned by the different predictive models. To provide a direct comparison of predictive performance, all selected tools, chosen based on their relevance, widespread use, and available information on model development, were evaluated on the same external OECD-derived dataset, and their results were compared with those obtained using LogPpred. In addition to the aforementioned tools, we included as an additional baseline the LogP values calculated with RDKit [27]. Although RDKit provides a calculation based on Crippen’s atom contribution method rather than a data-driven prediction, its extensive adoption in cheminformatics research makes its LogP estimate a relevant and practical reference baseline for comparison. As shown in Table 2, LogPpred achieved the best overall predictive performance among all evaluated tools on the external OECD-derived dataset, yielding both the lowest prediction error and the highest coefficient of determination. To assess the robustness of these performance estimates, 95% confidence intervals were calculated by bootstrap analysis based on 10,000 resampling iterations of the external dataset and are reported in Table 2 together with the corresponding MAE and R^2^ values. LogPpred showed an MAE of 0.84, with a bootstrap-derived 95% confidence interval of [0.76–0.91], and an R^2^ of 0.61, with a 95% confidence interval of [0.53–0.68]. Although Deep-PK achieved a comparable MAE (0.86 [0.76–0.94]), its lower R^2^ value (0.51) and confidence interval shifted toward lower values ([0.38–0.62]) indicate a reduced ability to reproduce the overall experimental LogP trends across the dataset. Additionally, a partial overlap between the Deep-PK training set and the OECD-derived external test set was identified, with 10 compounds found in common between the two datasets. This overlap should be taken into account when interpreting the predictive metrics obtained for Deep-PK, as it may lead to a partial overestimation of its performance on the OECD-derived external benchmark. Among the remaining predictors, SwissADME-XLogP3 (MAE = 0.95, R^2^ = 0.44), SwissADME-Consensus (MAE = 0.96, R^2^ = 0.56), VEGA-ALogP (MAE = 0.96, R^2^ = 0.47), and VEGA-MLogP (MAE = 0.98, R^2^ = 0.54) showed intermediate predictive performance. This trend was supported by their 95% confidence intervals, which showed MAE ranges shifted toward higher prediction errors compared with LogPpred, while the corresponding R^2^ intervals were generally lower than that of LogPpred and showed only limited overlap. In contrast, VEGA-Meylan/Kowwin, SwissADME-WLogP, SwissADME-Silicos-IT LogP, and SwissADME-ILogP displayed substantially higher prediction errors and markedly lower R^2^ values, indicating weaker agreement with the experimental data. This was further reflected by their confidence intervals, which remained shifted toward higher MAE values and lower R^2^ values compared with LogPpred. Furthermore, a partial overlap between the VEGA training set, which is common to all VEGA models considered in this study, and our external dataset was identified, with 12 compounds found in common, which may have affected the predictive metrics obtained for the VEGA models. For SwissADME models, the same overlap assessment could not be performed, as their datasets are not publicly available. By contrast, in the case of ADMET-AI, the training set was publicly available, but no overlap with the OECD-derived external benchmark was identified [28]. Overall, these results demonstrate that LogPpred provides the most accurate and reliable LogP estimates among the evaluated methods, highlighting the effectiveness of its descriptor-based ML framework for capturing complex structure–lipophilicity relationships.

Additionally, since the AD of LogPpred was defined and evaluated as described above, we further investigated whether the compared models provided an explicit domain of applicability assessment. To ensure a fair comparison under domain-aware conditions, we therefore recalculated the performance metrics of each model by considering only the compounds classified as falling within their respective domains of applicability. This analysis was not feasible for Deep-PK, ADMET-AI, RDKit and SwissADME models, as no explicit AD definition is provided. The results of this comparison are summarized in Table 3, where the predictive performance of LogPpred and the VEGA QSAR models is reported for compounds falling within their individual AD, along with the number of compounds retained within each model’s domain boundaries. As shown in Table 3, predictive performance metrics within the domain of applicability improve, as expected, compared to those calculated on the full external dataset (Table 2). Under these domain-aware conditions, LogPpred achieved the lowest prediction error (MAE = 0.62), while also retaining a substantial proportion of compounds within its defined domain. In contrast, all VEGA models displayed comparatively lower predictive robustness within their AD, as reflected by higher errors, and, in most cases, also a narrower domain breadth.

Overall, the superior estimation capability observed across a broad and independently sourced external dataset combined with a clearly defined AD that ensures wide chemical coverage and consistently strong in-domain robustness, underscores the reliability and practical applicability of LogPpred and supports its suitability for integration into regulatory frameworks and drug discovery workflows where accurate estimation of lipophilicity is essential for compound selection and prioritization.

### 2.4. Experimental Validation

**Figure 2:**
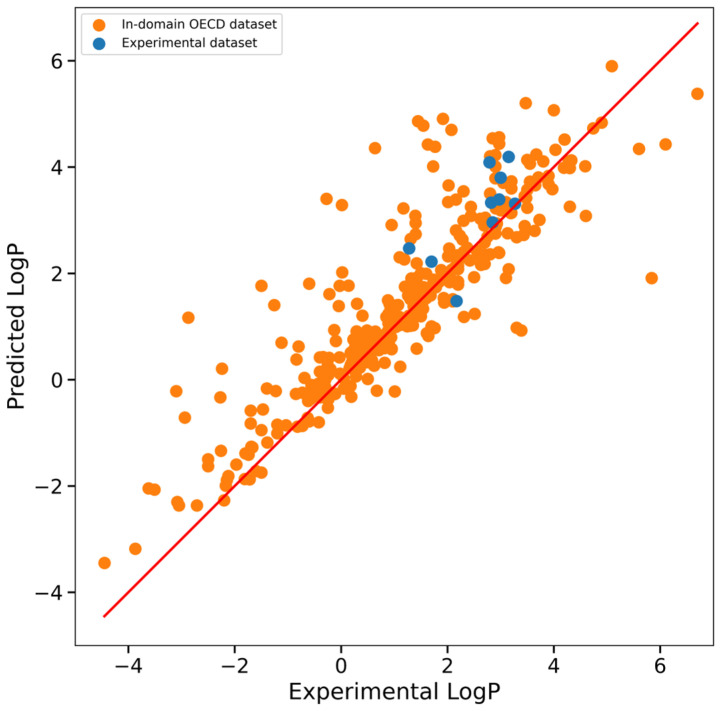
Experimental vs. predicted values for the 10 experimentally determined compounds (represented as blue dots) and in-domain OECD molecules (represented as orange dots).

As a further validation step for LogPpred, ten compounds were randomly selected from an in-house library and subjected to experimental LogP determination (Table S2). To better contextualize the structural relationship between these compounds and the training set, a nearest-neighbor similarity analysis based on Morgan fingerprints was also performed. Specifically, for each in-house compound, the Tanimoto similarity was computed against every molecule in the training set. For each compound, we considered the Tanimoto similarity with the most similar training-set molecule, and, to provide a more comprehensive assessment of the degree of structural similarity, the average Tanimoto similarity calculated over the 3, 5, and 10 closest neighbors. These values are reported in Table S3 of the Supporting Information. Overall, the maximum Tanimoto similarity with the closest training-set neighbor is relatively low for most compounds, generally around or below 0.45, indicating limited structural similarity to the training molecules. The only notable exception is compound M83, whose closest training-set neighbor exhibits a Tanimoto similarity of 0.73, suggesting a higher degree of structural resemblance while still representing a distinct chemical structure. Nevertheless, the average similarity for M83 decreases to 0.56, 0.51, and 0.43 when considering the 3, 5, and 10 nearest neighbors, respectively, indicating that this higher similarity is limited to a single close analogue rather than reflecting a broader cluster of highly similar training compounds. Similarly, for the remaining nine compounds, the average Tanimoto similarity over the 3, 5, and 10 nearest neighbors remains consistently low, ranging from approximately 0.27 to 0.44. Taken together, these results indicate that the 10 in-house compounds constitute a valuable external benchmark, challenging LogPpred with molecules that are, overall, structurally distinct from those used for training. Therefore, the LogP values were estimated for these in-house molecules using LogPpred and compared with the experimental measurements. Notably, all experimentally evaluated compounds fell within the AD of the model. As shown in Figure 2, the experimentally evaluated compounds closely followed the trend observed for the OECD dataset, showing good agreement between predicted and experimental LogP values. The model achieved an MAE of 0.66 on this external experimental set, confirming its ability to provide reliable lipophilicity estimates for previously unseen compounds. Although the prediction error was slightly higher than that observed during CV and on the internal test set, the overall predictive performance remained satisfactory. These findings further support the robustness and generalizability of LogPpred and demonstrate its applicability to structurally diverse molecules evaluated under real laboratory conditions. At the same time, given the limited size of the experimentally tested set, additional future studies on larger compound panels could further contribute to assessing the model’s performance.

### 2.5. Interpretability Analysis

To improve the interpretability of the developed LogPpred predictive model, a SHAP-based analysis was performed to provide a clearer understanding of the rationale underlying individual predictions and to reduce the black-box nature often associated with ML models. SHAP, which stands for SHapley Additive exPlanations, is a widely used explainable AI method based on Shapley values from cooperative game theory [29]. In this framework, each molecular descriptor can be interpreted as a “player” contributing to the final model output, and the SHAP value quantifies the extent to which that descriptor increases or decreases the predicted value for a given molecule. In the context of logP prediction, positive SHAP values indicate molecular features that contribute to increasing the predicted lipophilicity, whereas negative SHAP values indicate features that contribute to lowering the predicted logP. Therefore, this approach allows the model output to be decomposed into individual feature-level contributions, making it possible to identify which molecular descriptors mainly drive the prediction for a specific compound. This is particularly relevant for end users, as it supports a more transparent interpretation of the model response and provides chemically meaningful information that can assist the evaluation of predicted lipophilicity values.

To illustrate the practical application of this interpretability analysis, SHAP was applied to an exemplary compound selected from the 10 experimentally validated candidates, namely M336. The analysis was carried out using the model-agnostic Kernel SHAP method available in the SHAP Python library (v0.52.0), which estimates Shapley values through a local approximation strategy, allowing single-molecule predictions to be interpreted in a computationally feasible manner. The SHAP-based interpretation of the prediction is reported in Figure S2. In this plot, the descriptors with the strongest influence on the predicted logP value are highlighted. As expected, descriptors directly related to lipophilicity, such as *MolLogP* and *SlogP_VSA5*, were recognized by the model among the most relevant features contributing to the prediction. This finding supports the chemical consistency of the model, as RDKit descriptors encoding fragment-based logP contributions and regions associated with specific lipophilic properties were correctly identified as important determinants of the predicted value. Notably, however, the prediction was not driven exclusively by logP-related descriptors. Other features also contributed substantially, including descriptors associated with molecular polarity and hydrogen-bonding capacity. In particular, the absence of hydrophilic groups, as reflected by *NHOHCount* and *NumHDonors* values equal to zero, positively contributed to the predicted logP, consistently with the expected increase in lipophilicity for molecules lacking amine, alcohol, or other hydrogen-bond donor functionalities. In addition, *PEOE_VSA6* emerged as the most influential feature. *PEOE_VSA* descriptors encode the van der Waals surface area of atoms grouped according to their partial charges, with atomic partial charges estimated using the Partial Equalization of Orbital Electronegativities approach [30]. In this case, the high SHAP contribution associated with *PEOE_VSA6* indicates that this surface area descriptor based on partial charges played a major role in driving the model prediction. Overall, this example shows that the LogPpred output was guided by a combination of lipophilicity-related, polarity-related, and hydrogen-bonding descriptors, providing a chemically meaningful rationale for the predicted value, and exemplifying how the proposed interpretability workflow can be used to rationalize individual predictions. To make this analysis directly accessible to users, ultimately providing a more transparent view of the molecular features driving LogPpred outputs, the SHAP-based interpretability framework was implemented in a dedicated notebook available in the GitHub repository (https://github.com/MMVSL/LogPpred/blob/main/Interpretability_analysis.ipynb, accessed on 15 July 2026).

### 2.6. Comparison with State-of-the-Art LogP Datasets

As discussed above for the comparison with state-of-the-art prediction tools, the practical relevance of LogPpred should be evaluated not only in terms of its predictive performance, but also in relation to its potential integration into real drug-discovery pipelines. In the same way, when dataset construction represents a central component of model development, it is equally important to contextualize the dataset with respect to currently available public resources. This comparison is essential to clarify the coverage, novelty and specific contribution of the curated dataset used in this study, as well as to define its relationship with major and recently released LogP datasets. In particular, in the first half of 2026, two important public resources were released in the context of LogP data. The first is SangsterLogP, described by Cirino et al. [31] as the largest publicly available curated dataset of experimental LogP values and containing 23,520 unique compounds with annotated LogP measurements. The second dataset, herein referred to as GLP dataset, was instead reported by Grigorev et al. [32] and comprised 42,006 unique SMILES-LogP pairs compiled from public sources.

First, to compare the structural coverage of our curated dataset (comprising 13,536 unique molecules) with that of these recently released resources, we visualized the chemical space occupancy of the corresponding compounds by means of PCA. To ensure that the comparison was not dependent on a single molecular representation, the analysis was performed using three complementary fingerprint types, namely Morgan, RDKit, and PubChem fingerprints. As reported in Figure S3, projections showed a marked and consistent overlap between compounds from our dataset and those from SangsterLogP across all three fingerprint representations. The compounds from the two datasets occupied the same region of the projected chemical space, indicating that, despite its smaller size, our curated collection provides chemical space coverage broadly comparable to that of SangsterLogP. An analogous pattern was observed in the comparison with the GLP dataset, where PCA analysis again showed an extensive overlap across all three molecular representations (Figure S4). Taken together, these results indicate that, despite containing fewer compounds than SangsterLogP and GLP, the dataset developed in this study maintains a chemical space coverage comparable to that of larger public LogP resources.

The datasets were then compared in terms of property coverage, with particular attention to molecular weight, used as an additional indicator of compound representativeness, and to the experimental LogP range, which represents the target property and central focus of this study. In the comparison with SangsterLogP, our dataset covered a slightly narrower molecular weight range (~16–1200 Da) than SangsterLogP (~50–1660 Da), indicating that the latter includes a more extended coverage in terms of compound size. Interestingly, however, the comparison of experimental LogP values showed that our dataset spans a moderately wider lipophilicity interval, ranging from −5.08 to 12.18, whereas SangsterLogP ranges from −3.76 to 11.71. This broader experimental LogP coverage represents an element of novelty of the curated dataset compared to SangsterLogP, since highly hydrophilic and highly lipophilic compounds are particularly relevant for improving the representation of the outer regions of experimentally observed LogP space. A partially different trend was observed in the comparison with the GLP dataset. In this case, the experimental LogP interval covered by GLP was slightly broader overall, extending from −9.60 to 9.98 (compared to our ranging from −5.08 to 12.18). Nevertheless, it is worth mentioning that the two ranges are not fully superimposable, since GLP included compounds with lower LogP values, while our dataset extended further toward highly lipophilic compounds. Therefore, although GLP provides broader coverage on the hydrophilic side, our dataset contributes complementary coverage in the high-LogP region, further reinforcing its added value within the landscape of currently available public LogP datasets. This complementarity was also supported by the comparison of molecular weight ranges. While GLP covered an approximate interval of 80–850 Da, our dataset extended from approximately 16 to 1200 Da, thus including both smaller and larger molecules not represented in the same range by GLP. Overall, these analyses indicate that the curated dataset presented in this study should not be regarded simply as a smaller alternative to recently released public LogP datasets. Rather, it represents a complementary resource that covers a broadly comparable chemical space while contributing additional coverage in specific regions of physicochemical property space, particularly in terms of LogP range with respect to SangsterLogP and molecular-weight range and high-LogP coverage with respect to GLP. In light of this complementary value, the curated dataset has been made publicly available through the GitHub repository associated with this study, with the aim of facilitating reuse by the scientific community, including its application as an external benchmark for future LogP prediction models.

To further broaden the contextualization of the present dataset, other less recent but widely used lipophilicity-related resources, such as MoleculeNet [33], EPI Suite, and OpenChem [34], should also be considered. However, these resources differ in scope and practical accessibility. For example, the MoleculeNet lipophilicity dataset is based on logD values rather than experimental LogP measurements. Similarly, the US EPA EPI Suite is based on the PHYSPROP database which, despite representing an important source of physicochemical property data, is not directly accessible through the software interface. In contrast, OpenChem provides an easily accessible LogP dataset and was therefore further examined in comparison with our curated collection. Analogously to the comparisons performed with SangsterLogP and GLP, the chemical space coverage of the two datasets was evaluated by PCA (Figure S5), which again showed a marked overlap between compounds from our dataset and those from OpenChem. The comparison was then extended to property coverage by analyzing molecular weight and experimental LogP ranges, following the same strategy previously adopted. Although the LogP intervals covered by the two datasets were comparable, with OpenChem ranging from −5.40 to 11.29, our curated collection showed a broader molecular weight range (~16–1200 Da) compared with OpenChem, which approximately spans from 42 to 916 Da. This trend, already observed in the comparison with GLP, further supports the added value of the present dataset as a complementary and openly accessible resource for LogP curated data. Furthermore, to provide readers with a clearer quantitative overview of the relationships between each public dataset and our curated dataset, structural duplicates within each dataset and compounds overlapping with our dataset were identified based on Tanimoto similarity calculated using Morgan fingerprints. The resulting information is summarized in Table S4, which reports, for each public resource considered, the original dataset size, the number of unique compounds remaining after structural deduplication, the number of compounds shared with our curated dataset, and the numbers of compounds exclusively present in either the public resource or our dataset.

In addition, recognizing that these resources represent not only state-of-the-art available datasets but also important reference benchmarks for lipophilicity prediction, they were also used to further evaluate the predictive behavior of LogPpred. To this end, compounds shared between each benchmark dataset and our curated pool were first identified and removed, in order to avoid any overlap with the data used for model development. LogPpred predictions were then generated for the remaining compounds from each external resource, and each compound was assigned to the in-domain or out-domain category according to the AD assessment implemented in LogPpred, allowing this additional external evaluation to account not only for prediction accuracy but also for the representativeness of each compound with respect to the model domain. As a result, for SangsterLogP, 6740 compounds were classified as in-domain, and LogPpred achieved an MAE of 0.55 on this subset. For the GLP dataset, 18,289 compounds were classified as in-domain, with an MAE of 0.48. Finally, for OpenChem, 1983 compounds were classified as in-domain, and the corresponding predictions showed an MAE of 0.49. Notably, these MAE values are consistent with, and even lower than, those previously obtained in the external validation performed on the experimental and OECD datasets. Taken together, these results underscore the capability of LogPpred to retain strong predictive performance within its domain across three additional recently released and independent public LogP benchmarks, further supporting the practical value of the herein proposed model which consistently shows robust predictive behavior on external data. Overall, this analysis, which combined chemical space coverage comparison, property range evaluation, and performance benchmarking, allowed us not only to further validate LogPpred on additional state-of-the-art datasets but also to better define the contribution of the curated dataset developed in this study. The results show that, despite its smaller size compared with recently released resources, the dataset provides comparable chemical space coverage, distinctive structural and LogP range profiles, and strong external benchmark performance, thereby introducing novel elements that establish it as a valuable and complementary data repository in the current landscape of publicly available LogP resources. Furthermore, as discussed by Fritschka and Sadowski [28], experimental LogP measurements are affected by several intrinsic sources of uncertainty that limit the achievable agreement between independent measurements. These include the mutual solubility of water and octanol, deviations from ideal partitioning behavior, concentration-dependent effects, partial ionization of weak acids and bases, and methodological differences among experimental protocols. Consequently, even carefully curated datasets inevitably contain a level of experimental variability that cannot be eliminated computationally. Therefore, the predictive performance achieved by LogPpred, particularly on fully independent external datasets, approaches the practical accuracy limit imposed by the uncertainty of the underlying experimental reference data. Under these conditions, further improvements in prediction accuracy are expected to depend primarily on the availability of more accurate and consistent experimental measurements rather than on increasingly sophisticated machine learning architectures.

## 3. Materials and Methods

### 3.1. Data Set Curation

An extensive manual literature search was conducted on PubMed, resulting in the collection of 18,806 molecules with reported experimental LogP values derived from 110 PubMed-indexed articles; these data subsequently underwent a standardized curation workflow, as detailed hereafter. Firstly, all structures were canonicalized using the RDKit Python library (v2024.3.5) [27], after which salts and invalid entries were removed. Subsequently, structural duplicates were removed through a hierarchical filtering procedure based on multiple molecular representations. For each representation, namely Morgan, PubChem, RDKit fingerprints and RDKit physicochemical descriptors, the dataset was scanned to identify identical entries (duplicates), meaning that their fingerprints produced a Tanimoto similarity equal to 1. In the case of RDKit descriptors, identity was defined as a Euclidean distance of 0, which indicates that all descriptor values matched perfectly. When two or more identical entries showed different experimental LogP values, they were removed to eliminate conflicting measurements. When the reported LogP values were consistent, only a single representative entry was retained. This procedure ensured that the final dataset contained only unique compound–LogP pairs consistently defined across all representations. After curation, the final dataset consisted of 13,536 unique molecules, which was randomly split into an 80/20 training/test partition. Importantly, the duplicate removal procedure also prevented any form of train–test leakage. In addition, an independent external test set was assembled for validation purposes. A total of 858 molecules with experimentally reported LogP values were retrieved from OECD eChemPortal, a regulatory database that aggregates data produced under standardized testing guidelines. Specifically, entries measured according to OECD Test No. 107 were collected. These entries underwent the same curation workflow applied to the primary dataset, including structural standardization, removal of invalid molecules and hierarchical duplicate filtering. To ensure that the external set contained only compounds that were fully distinct from those used for model development, the duplicate identification procedure was also applied across the two datasets. All molecules that were found to be identical to any compound in the primary dataset, according to any of the molecular representations employed, were removed. This process resulted in a curated set comprising 652 unique molecules used exclusively for external evaluation.

### 3.2. Molecular Representations

#### 3.2.1. Descriptor- and Fingerprint-Based Representations

Two families of molecular representations were considered in this work: binary encodings, referred to as FPs, and nonbinary encodings, derived from physicochemical and structural descriptors. Consistent with previous studies, these approaches were selected due to their demonstrated effectiveness in capturing relevant chemical information for predictive modeling tasks [35]. All representations were computed using the RDKit Python library functionalities [27].

*Morgan FPs* encode local atomic environments within a radius of two bonds around each heavy atom; the neighborhood information is hashed into identifiers, producing a 1024-bit binary vector.

*RDKit FPs* capture atom-bond connectivity patterns, representing subgraph-based structural features through hashed 1024-bit vectors.

*PubChem FPs* consist of 881 predefined structural keys derived from a curated dictionary of molecular fragments, with each bit indicating the presence or absence of a specific substructure.

*RDKit Descriptors* provide continuous numerical information describing physicochemical and topological properties. A total of 208 descriptors were computed for each molecule. Compared with their 3D counterparts, these descriptors provide a less computationally demanding and more directly reproducible molecular representation, as they do not require conformer generation or geometry optimization. The descriptors values were scaled using a normalization approach implemented in the Python Scikit-learn library.

#### 3.2.2. ChemBERTa and GNN Embeddings

In addition to descriptor- and fingerprint-based encodings, two learned molecular representations based on deep learning were employed: a sequence-based embedding derived from ChemBERTa and a graph-based embedding obtained using a cGNN. Unlike handcrafted representations, these approaches learn task-relevant molecular features directly from raw molecular encodings, enabling the implicit capture of complex structural and physicochemical patterns. For ChemBERTa, canonical SMILES strings were used as input and processed using the pretrained deepchem/ChemBERTa-77M-MTR model, a RoBERTa-based molecular language model trained on approximately 77 million compounds. For each molecule, the final hidden state of the prepended classification token, referred to as [CLS], was extracted as a fixed-length representation, providing a global summary of the input sequence for downstream tasks. For the GNN-based representation, molecules were converted into graph structures using RDKit, where nodes correspond to atoms and edges represent covalent bonds. Atom-level features and bond connectivity information were processed using a cGNN implemented in PyTorch (https://pytorch.org/, accessed on 15 July 2026). Message-passing layers were applied to iteratively update node representations, followed by a global pooling operation to obtain fixed-length molecular embeddings.

### 3.3. Machine Learning Algorithms

Five traditional supervised regression algorithms were employed to develop predictive models for LogP estimation: RFR, SVR, KNR, GPR, and MLP. All models were implemented using the dedicated functions available in the Scikit-learn Python library [36] and subjected to hyperparameter optimization using the GridSearch approach, as detailed in the following section.

RFR is an ensemble learning algorithm composed of multiple decision trees, each producing an individual prediction. The final output is obtained by averaging predictions across all trees. The main hyperparameters optimized in this study were n_estimators (number of trees, with tested values being 200 and 700), max_features (fraction of features considered at each split, with tested values being None, “sqrt” and “log2”), and max_depth (maximum tree depth, with tested values being 5, 15, 30, None).

SVR performs nonlinear regression by mapping input data into a high-dimensional space through a kernel function and identifying the hyperplane that minimizes prediction error within a defined margin. Among the main hyperparameters optimized were the kernel type (linear, polynomial, or radial basis function) and the regularization parameter C, which governs the balance between margin width and training error (evaluated over values of 0.01, 0.1, 1, 10 and 100).

KNR predicts the output of a query compound based on the average value of its k closest neighbors in feature space. Hyperparameters optimized included n_neighbors (with tested values being odd numbers ranging from 3 to 15) and weights, which determine whether all neighbors contribute equally or with distance-dependent influence.

GPR is a probabilistic nonlinear regression method that defines a distribution over functions and performs inference using a kernel-based covariance structure. Kernel selection served as the primary optimization step, as it directly impacts model smoothness, flexibility, and noise handling. In particular, RBF, RationalQuadratic, DotProduct, Matern and WhiteKernel implementations available in scikit-learn library were evaluated during hyperparameter optimization.

MLP is a feed-forward artificial neural network trained through backpropagation. The hyperparameters optimized included hidden_layer_sizes (number and size of hidden layers, with tested combinations being 100–100; 100–1000 and 100–200–1000), activation function (logistic, tanh, or ReLU), and learning_rate_init (optimized over values of 0.01, 0.001 and 0.0001). Additional training parameters, such as max_iter and early_stopping, were used to prevent overfitting and ensure convergence stability.

In addition to traditional regression algorithms, deep learning models were implemented: a transformer-based model (ChemBERTa) and a cGNN.

The ChemBERTa-based model uses canonical SMILES strings as input and relies on a pretrained transformer architecture, which is fine-tuned in an end-to-end supervised regression setting. A regression head was appended to the transformer to predict molecular lipophilicity (LogP). To mitigate overfitting and ensure training stability, early stopping based on validation set performance was applied. The model was trained for a maximum of 100 epochs and training was halted if no improvement in validation MAE greater than 1 × 10^−4^ was observed for five consecutive epochs.

The cGNN uses molecular graph representations derived from canonical SMILES strings. In this approach, SMILES strings were first converted into molecular graphs, which were subsequently processed by the cGNN to generate fixed-length molecular embeddings through message-passing operations. These learned embeddings were used as input features for an MLP neural network, trained in a supervised regression setting. Hyperparameter optimization was performed using the Optuna Python library, and an Optuna-based pruning strategy was employed. Early stopping based on validation performance was applied, with a maximum of 200 training epochs.

### 3.4. Model Building and Evaluation

By combining descriptor- and fingerprint-based molecular representations with the five traditional ML algorithms, 20 predictive models were constructed. Deep learning models based on ChemBERTa and a cGNN were additionally developed.

For the traditional ML models, hyperparameter optimization was performed using Scikit-learn’s *GridSearchCV* and was nested within the validation procedure. Specifically, we performed 10 independent random 70:30 partitions of the training set. For each repetition, the training partition (70%) was used to identify the optimal hyperparameter configuration by means of an internal 5-fold *GridSearchCV*, after which the resulting model was evaluated on the corresponding validation partition (30%). The final hyperparameter configuration was selected as the one identified as optimal in the highest number of repetitions. Random data partitions were generated using the *train_test_split* function from Scikit-learn’s *model_selection* module, with the random_state set to *iteration number × 50*. For ChemBERTa, no hyperparameter optimization was performed; the model was evaluated using the same 10 independent 70:30 random partitions adopted for the traditional ML models.

On the other hand, for the cGNN model hyperparameter optimization was performed once using Optuna prior to model evaluation across different data splits. The training set was initially divided into a training partition (90%) and a validation partition (10%), which were used by Optuna to identify the optimal hyperparameter configuration. The selected hyperparameters were then kept fixed and model performance was evaluated across different data splits using a standard 10-fold cross-validation (CV) implemented through Scikit-learn’s *KFold* function with *random_state = 25*.

Subsequently, all optimized models were re-trained on the entire training set using the hyperparameter setting previously identified, and tested on the hold-out independent test set, which was not used during model training or optimization. Predictive performance was quantified using MAE, calculated according to:

(1) $$MAE=\frac{1}{n}\sum_{i=1}^{n}|y_{i}-{\hat{y}}_{i}|$$

where $y_{i}$ denotes the experimental values, ${\hat{y}}_{i}$ the predicted outputs, and n represents the total number of observations. MAE (1) scores fall in the range (0; +∞), where 0 indicates perfect predictions.

To further quantify prediction error, the deviation between predicted and true values was measured using the coefficient of determination (R^2^), defined as:

(2) $$R^{2}=1-\frac{\sum_{i=1}^{n}(y_{i}-{\hat{y}}_{i}{)}^{2}}{\sum_{i=1}^{n}(y_{i}-\bar{y}{)}^{2}}$$

where $y_{i}$ denotes the experimental values, ${\hat{y}}_{i}$ the predicted outputs, and $\bar{y}$ the mean of the experimental data. R^2^ (2) values fall in the range (−∞, +1). R^2^ values equal to 1 indicate perfect agreement between predicted and experimental values, whereas values close to 0 or negative indicate limited or no predictive power.

### 3.5. Applicability Domain Assessment

Model reliability was evaluated through the definition of the AD, which identifies the chemical space where predictions can be considered reliable. The AD of the best-performing model was determined using the ADvisor tool [22], a model-agnostic framework that enables the systematic evaluation and optimization of multiple AD strategies. The explored approaches spanned different methodological families, including distance-based methods (e.g., distance-to-neighbors, DB, and leverage, LEV), distribution-based approaches (e.g., kernel density estimation, KDE), fragment-based methods (FB), geometric approaches based on PCA, ML-based methods (e.g., one-class SVM, OCSVM), and composite strategies. These approaches rely on complementary principles, such as structural similarity, statistical distribution of the training data, or the presence of known molecular fragments, to assess whether a query compound falls within the model domain. The training and test sets, including canonical SMILES, experimental values, and model predictions, were provided as input, as required by the tool. The optimal AD strategy was selected based on its ability to discriminate between reliable and unreliable predictions by comparing prediction errors for in-domain and out-of-domain compounds. Strategies were prioritized when higher prediction errors were observed outside the domain compared to inside, while maintaining adequate coverage of the data. The selected definition was subsequently applied to the external set, enabling the classification of each compound as either *in-domain* or *out-of-domain*.

### 3.6. Experimental Determination

Partition coefficients between n-octanol and phosphate-buffered saline (PBS) at pH 7.4 were determined using the traditional shake-flask method at room temperature [37,38]. Before the experiments, the organic phase (n-octanol) and the aqueous phase (12 mM PBS, 137 mM NaCl, 2.7 mM KCl, pH 7.4) were mutually saturated by agitating the mixture for at least 4 h. Test compounds were dissolved in pre-saturated PBS containing 1% DMSO at the highest concentration permitted by their thermodynamic solubility. After adding appropriate volumes of pre-saturated n-octanol, samples were shaken at 250 rpm for 45 min to ensure partitioning equilibrium. The samples were then separated by centrifugation at 3000 rpm for 15 min. The concentration of the remaining solute in the aqueous phase was quantified by UV-Vis spectrophotometry using a Cary 50 UV–Vis spectrophotometer (Varian, Inc., Palo Alto, CA, USA). Absorbance values were recorded at the maximum wavelength for each compound and interpolated using standard calibration curves prepared in the same matrix (saturated PBS with 1% DMSO). Each reported value was calculated as the mean of at least six independent measurements. For the compounds investigated, no significant ionization is expected under the experimental conditions (pH 7.4), as supported by their structural features and predicted ionization states [39]; therefore, the measured distribution coefficients (logD_7.4_) can be considered representative of their partition coefficients (logP).

## 4. Conclusions

In this work, we developed LogPpred, an ML model designed to provide accurate and reliable predictions of molecular lipophilicity, expressed as the octanol/water partition coefficient (LogP). Using a large collection of experimentally determined LogP values, a rigorous data curation workflow was applied to ensure structural consistency, remove duplicates, and assemble a high-quality dataset suitable for the development of predictive models. Multiple molecular representations and five ML algorithms were systematically explored, yielding 22 model configurations optimized through an extensive CV procedure. Across all tested approaches, molecular descriptors proved to be the most informative representation, and the combination of GPR with RDKit descriptors consistently outperformed the alternatives. This model, designated LogPpred, demonstrated excellent predictive accuracy on both the internal test set and an independent external dataset, confirming its ability to generalize across structurally diverse compounds. The results obtained in external evaluation further demonstrated the strong predictive performance of LogPpred relative to several widely used LogP predictors and calculators based on fragmental, atomistic, and deep-learning methodologies. Notably, LogPpred achieved both the lowest prediction error and the highest correlation with experimental values among the evaluated methods. Moreover, to enhance its interpretability and support its responsible use, the AD of LogPpred was defined using the ADvisor tool. The selected ComAD strategy effectively distinguished reliable predictions within the learned chemical space from less certain extrapolations, providing an additional layer of confidence for downstream decision-making. Notably, the performance of LogPpred within its applicability domain surpassed that of the other models evaluated within their respective domains, while maintaining broad chemical coverage and a substantial proportion of in-domain compounds. Remarkably, considering all the external evaluations performed in this study, LogPpred was validated on a total of five independent external datasets, including the experimental validation set, the OECD dataset, and three public LogP benchmark datasets. Across these diverse external resources, the model consistently showed strong predictive performance, further supporting its reliability and generalizability. Overall, these results demonstrate that LogPpred is a robust, transparent, and accurate tool for the estimation of molecular lipophilicity, thereby representing a valuable computational resource that can support compound prioritization, reduce experimental workload, and accelerate decision-making in modern drug discovery. Importantly, the curated dataset developed herein represents a further asset of this study, providing additional value as a complementary data repository in the context of publicly available LogP resources. To promote transparency, reproducibility, and ease of use, LogPpred is made openly available to the scientific community through a dedicated GitHub repository (https://github.com/MMVSL/LogPpred, accessed on 15 July 2026), enabling straightforward integration into a wide range of research workflows.

## Figures and Tables

**Table 1:** Performance evaluation results based on test set prediction.

Model	MAE	R^2^
GPR descriptors	0.34	0.92
SVR descriptors	0.35	0.91
MLP descriptors	0.39	0.90
SVR PubChem	0.43	0.88
RFR descriptors	0.45	0.87
ChemBERTa-77M-MTR	0.46	0.88
KNR descriptors	0.49	0.85
MLP PubChem	0.50	0.85
SVR Morgan	0.51	0.83
RFR PubChem	0.55	0.81
MLP Morgan	0.57	0.81
SVR RDKit	0.57	0.79
MLP RDKit	0.63	0.75
GPR PubChem	0.64	0.77
cGNN	0.65	0.73
KNR PubChem	0.67	0.72
RFR Morgan	0.71	0.70
KNR RDKit	0.72	0.69
RFR RDKit	0.72	0.69
GPR Morgan	0.74	0.70
KNR Morgan	0.79	0.61
GPR RDKit	2.22	−1.27

**Table 2:** Predictive performance of LogPpred compared with established LogP prediction tools. Values in square brackets indicate the bootstrap-derived 95% confidence interval for each metric.

Tool	MAE	R^2^
LogPpred	0.84 [0.76–0.91]	0.61 [0.53–0.68]
Deep-PK *	0.86 [0.76–0.94]	0.51 [0.38–0.62]
SwissADME-XLogP3 **	0.95 [0.86–1.04]	0.44 [0.30–0.56]
SwissADME-Consensus **	0.96 [0.89–1.03]	0.56 [0.48–0.63]
VEGA–AlogP *	0.96 [0.87–1.04]	0.47 [0.35–0.57]
VEGA–MlogP *	0.98 [0.90–1.05]	0.54 [0.47–0.61]
SwissADME-MlogP **	0.98 [0.90–1.06]	0.52 [0.44–0.59]
RDkit calculated LogP	1.02 [0.93–1.11]	0.43 [0.29–0.55]
ADMET-AI	1.02 [0.93–1.11]	0.43 [0.29–0.55]
VEGA–Meylan/Kowwin *	1.12 [1.02–1.23]	0.25 [0.02–0.44]
SwissADME-WlogP **	1.21 [1.10–1.32]	0.16 [−0.07–0.34]
SwissADME-Silicos-IT LogP **	1.24 [1.15–1.33]	0.32 [0.18–0.44]
SwissADME-IlogP **	1.38 [1.29–1.48]	0.17 [−0.11–0.34]

* Partial overlap with the OECD-derived external benchmark was identified in the publicly available training set of this tool. ** The training set used by this tool is not publicly disclosed; therefore, a possible overlap with the OECD-derived external benchmark cannot be excluded.

**Table 3:** Predictive performance of LogPpred and VEGA models evaluated within their respective AD.

Tool ^1^	In-Domain MAE	Number of In-Domain Compounds
LogPpred	0.62	342
VEGA–ALogP	0.68	336
VEGA–Meylan/Kowwin	0.72	262
VEGA–MLogP	0.77	308

^1^ Deep-PK, ADMET-AI, SwissADME and RDKit calculated LogP were not included in this analysis because no explicit AD definition is provided for these methods.

## Data Availability

The logPpred ML model was made publicly available as a predictive tool on GitHub (https://github.com/MMVSL/LogPpred, accessed on 15 June 2026). Additional data useful for reproducibility, including the curated dataset comprising 13,536 instances, can be accessed through the link indicated in the dedicated section of the GitHub repository, which is also reported here: http://www.mmvsl.it/wp/wp-content/uploads/2026/07/Reproducibility.zip (accessed on 15 July 2026).

## Supplementary Materials

The following supporting information can be downloaded at https://www.mdpi.com/article/10.3390/molecules31152566/s1, Figure S1: PCA of training and test sets. Table S1: Cross validation results for the developed models. Table S2: Experimental and calculated LogP of the third external data set. Table S3: Similarity analysis between the experimentally tested compounds and the training set. Figure S2: Feature importance analysis for compound M336. Figure S3: PCA of our curated dataset and SangsterLogP dataset. Figure S4: PCA of our curated dataset and GLP dataset. Figure S5: PCA of our curated dataset and OpenChem dataset. Table S4: Quantitative comparison of the present curated dataset with public LogP datasets.

## Abbreviations

The following abbreviations are used in this manuscript:

ML	Machine Learning
AD	applicability domain
PCA	principal component analysis
cGNN	convolutional graph neural network
FP	fingerprint
RFR	Random Forest Regressor
SVR	Support Vector Regression
KNR	K-Nearest Neighbors Regressor
GPR	Gaussian Process Regression
MLP	Multilayer Perceptron
MAE	mean absolute error
AI	artificial intelligence
CV	cross-validation
SMILES	Simplified Molecular Input Line Entry System
OECD	Organisation for Economic Co-operation and Development
PBS	Phosphate-Buffered Saline
