# Supplementary material for: LogPpred: An AI-Based Predictive Model for Accurate Estimation of Molecular LogP
Source: Molecules. 2026 Jul 23;31(15):2566. doi: 10.3390/molecules31152566 (PMC13468394; doi:10.3390/molecules31152566)
Supplement: Supplementary file 1 [file molecules-31-02566-s001.zip › molecules-4414570-supplementary.pdf]

LogPpred: An AI-Based Predictive Model for Accurate Estimation of Molecular LogP

Lisa Piazza[1], Lara Sortino[1], Alessio Costa[1,2], Clarissa Poles[3], Federico Fornaseri[4], Stefano Sainas[4], Marta Giorgis[4], Giulio Poli[1], Marco Macchia[1], Marco L. Lolli[4], Tiziano Tuccinardi[1,5] and Miriana Di Stefano[1,5]

1 Department of Pharmacy, University of Pisa, 56126 Pisa, Italy
2 Department of Life Sciences, University of Siena, 53100 Siena, Italy
3 Genomics and Experimental Medicine Program, Scuola Superiore Meridionale (SSM, School of Advanced Studies), Via Mezzocannone 4, 80078 Napoli, Italy
4 Department of Drug Science and Technology, University of Turin, via Pietro Giuria 9, Turin, 10125, Italy
5 Consorzio Interuniversitario Nazionale per la Scienza e Tecnologia dei Materiali (INSTM), 50121 Firenze, Italy
* Correspondence: tiziano.tuccinardi@unipi.it

Figure S1. PCA of training and test sets S2

Table S1. Cross validation results for the developed models S2

Table S2. Experimental and calculated LogP of the third external data set S3

Table S3. Similarity analysis between the experimentally tested compounds and the training set S5

Figure S2. Feature importance analysis for compound M336 S5

Figure S3. PCA of our curated dataset and SangsterLogP dataset S6

Figure S4. PCA of our curated dataset and GLP dataset S6

Figure S5. PCA of our curated dataset and OpenChem dataset S6

Table S4. Quantitative comparison of the present curated dataset with public LogP datasets S7

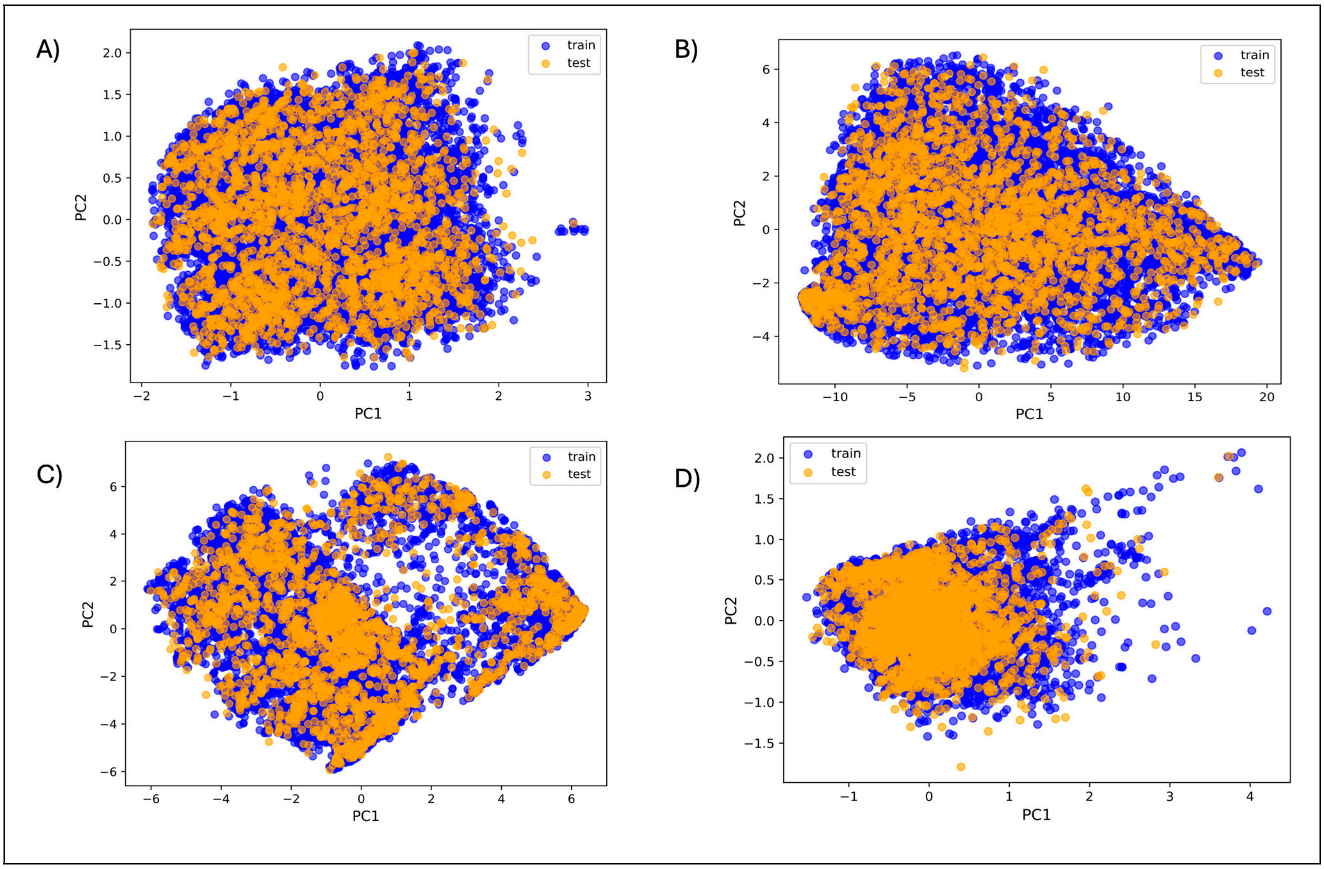

Figure S1. Principal component analysis (PCA) performed for training and test sets using Morgan fingerprints (a), RDKit fingerprints (b), PubChem fingerprints (c), and molecular descriptors (d).

Table S1. Evaluation results, expressed in terms of MAE, obtained for the developed models through cross-validation analysis.

Model	CV MAE
GPR_descriptors	0.34 ± 0.01
SVR_descriptors	0.38 ± 0.01
MLP_descriptors	0.44 ± 0.01
RFR_descriptors	0.48 ± 0.01
ChemBERTa-77M-MTR	0.50 ± 0.03
SVR_Morgan	0.55 ± 0.01
RFR_PubChem	0.60 ± 0.02
SVR_PubChem	0.62 ± 0.01
GPR_PubChem	0.63 ± 0.01
SVR_RDKit	0.64 ± 0.01
KNR_descriptors	0.65 ± 0.01
KNR_PubChem	0.69 ± 0.01
MLP_PubChem	0.70 ± 0.01
GPR_RDKit	0.72 ± 0.01

KNR_RDKit	0.72 ± 0.01
MLP_RDKit	0.74 ± 0.01
GPR_Morgan	0.74 ± 0.01
RFR_Morgan	0.74 ± 0.01
RFR_RDKit	0.75 ± 0.01
cGNN	0.76 ± 0.01
KNR_Morgan	0.79 ± 0.01
MLP_Morgan	0.82 ± 0.02

Table S2. Experimental and calculated LogP of the third external data set.

ID	Mol	Exp. LogP	LogPpred
**M49**	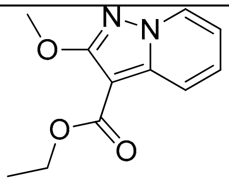	2.17	1.48
**M124**	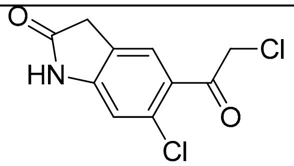	1.70	2.22
**M263**	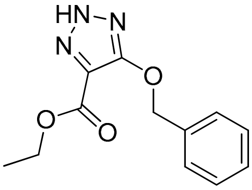	1.28	2.47
**M336**	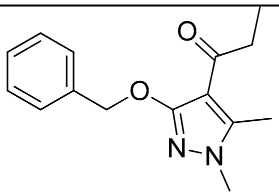	2.85	2.96
**M83**	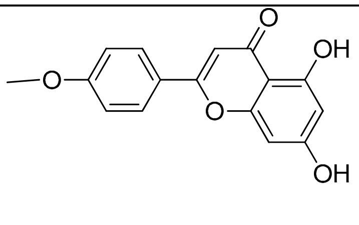	3.27	3.31

**M13**	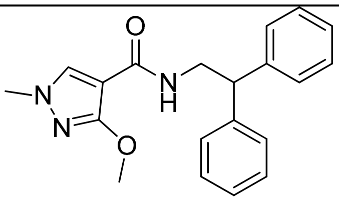	2.82	3.33
**M509**	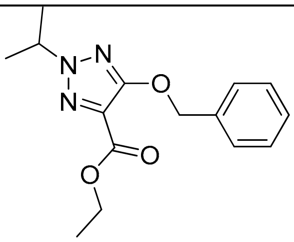	2.97	3.39
**M513**	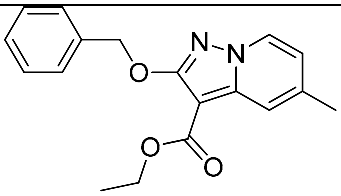	3.00	3.8
**M418**	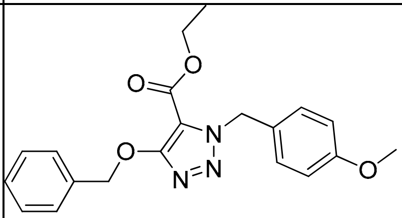	2.79	4.09
**M773**	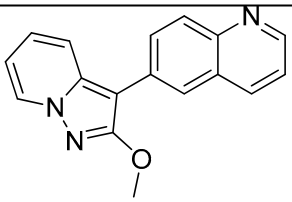	3.15	4.19

Table S3. Structural similarity between the experimentally tested compounds and the training set. Structural similarity was assessed using the Tanimoto similarity (TN) computed on Morgan fingerprints. Max TN corresponds to the Tanimoto similarity with the closest training-set neighbor, whereas for the top 3, top 5, and top 10 nearest neighbors, the mean Tanimoto similarity (Mean TN) and the corresponding standard deviation (Std) are reported.

Compound ID	Max TN	Mean TN (top 3)	Std (top 3)	Mean TN (top 5)	Std (top 5)	Mean TN (top 10)	Std (top 10)
**M49**	0.42	0.41	0.01	0.40	0.01	0.40	0.01
**M124**	0.35	0.31	0.04	0.29	0.04	0.27	0.03
**M263**	0.45	0.44	0.01	0.43	0.02	0.42	0.01
**M336**	0.39	0.37	0.02	0.36	0.02	0.35	0.02
**M83**	0.73	0.56	0.15	0.51	0.12	0.43	0.12
**M13**	0.35	0.34	0.01	0.34	0.01	0.33	0.01
**M509**	0.46	0.44	0.02	0.43	0.02	0.41	0.02
**M513**	0.41	0.38	0.02	0.37	0.02	0.36	0.02
**M418**	0.45	0.41	0.04	0.40	0.03	0.39	0.02
**M773**	0.45	0.38	0.06	0.37	0.04	0.35	0.04

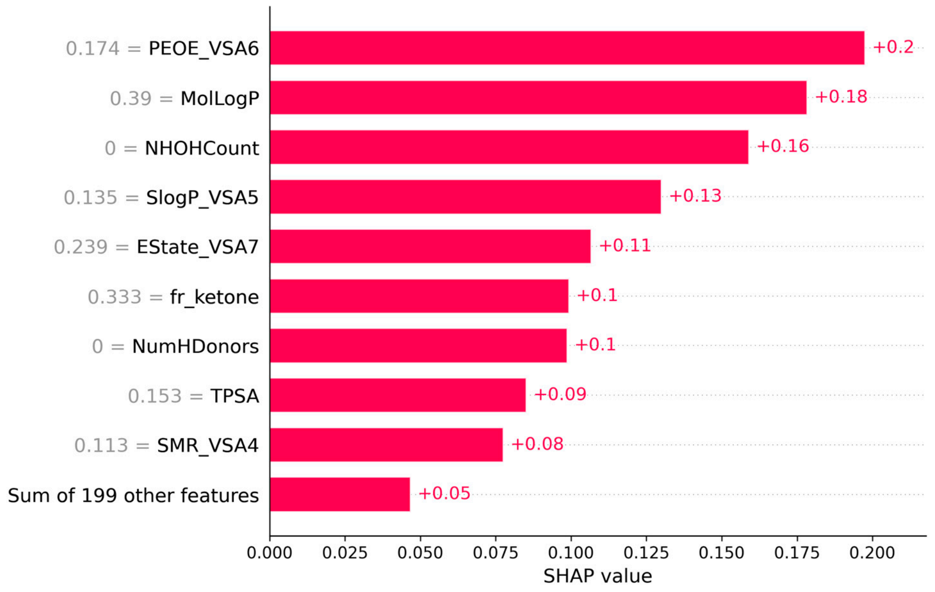

Figure S2. Feature importance analysis for compound M336 based on SHAP interpretation.

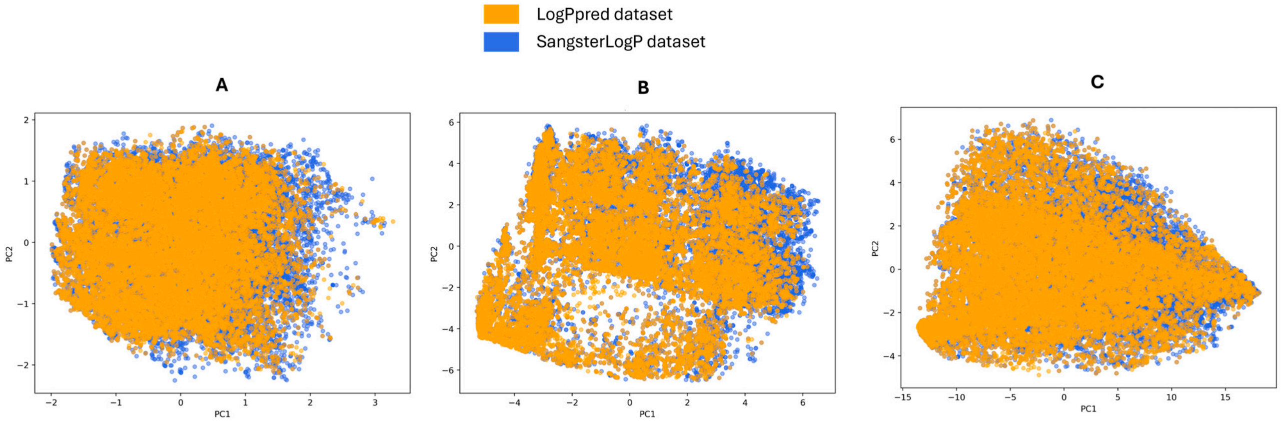

Figure S3. PCA of our curated dataset (represented by orange dots) and SangsterLogP dataset (represented by blue dots) using Morgan fingerprints (A), PubChem fingerprints (B) and RDKit fingerprints (C).

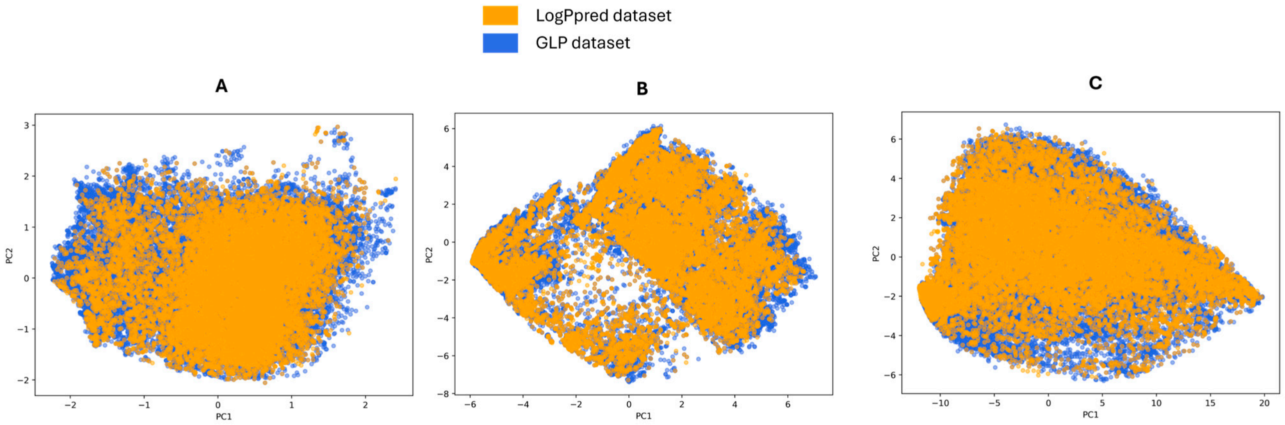

Figure S4. PCA of our curated dataset (represented by orange dots) and GLP dataset (represented by blue dots) using Morgan fingerprints (A), PubChem fingerprints (B) and RDKit fingerprints (C).

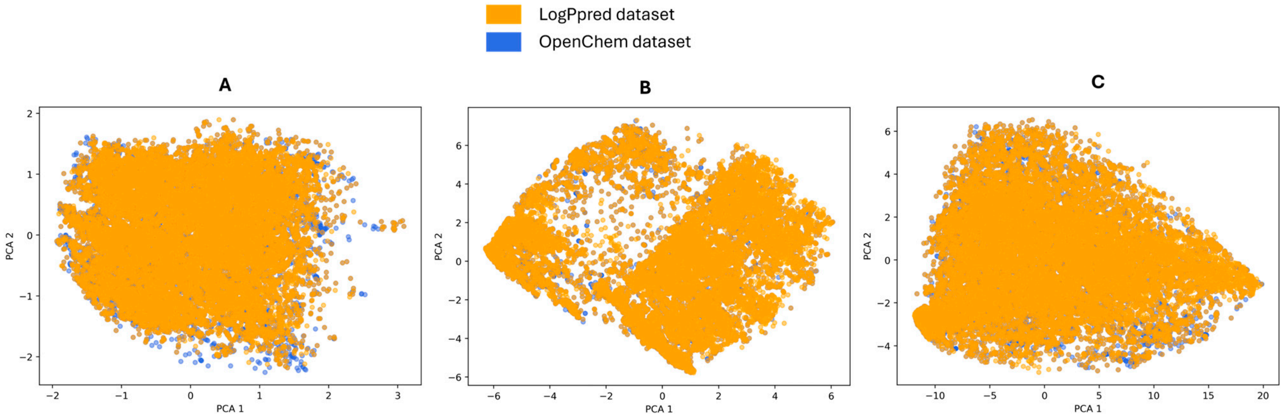

Figure S5. PCA of our curated dataset (represented by orange dots) and OpenChem dataset (represented by blue dots) using Morgan fingerprints (A), PubChem fingerprints (B) and RDKit fingerprints (C).

Table S4. Quantitative comparison of the present curated dataset with public LogP datasets. “Total” indicates the original number of compounds in each public dataset, whereas “Unique” indicates the number of compounds remaining after structural deduplication based on Tanimoto similarity calculated using Morgan fingerprints. “Overlapping” reports the number of compounds shared with the LogPpred dataset. “Only in dataset” and “Only in LogPpred dataset” indicate the numbers of compounds exclusively present in the public dataset and in the LogPpred dataset, respectively.

Dataset	Total	Unique	Overlapping	Only in dataset	Only in LogPpred dataset
**SangsterLogP**	23520	23040	10238	12802	3298
**GLP**	42006	40894	9950	30944	3586
**OpenChem**	14176	13887	11273	2614	2263
